# Biology, Antioxidant Activity, and Therapeutic Potential of *Cistus* sp.—A Comprehensive Review

**DOI:** 10.3390/ijms26136400

**Published:** 2025-07-03

**Authors:** Patrycja Kielar, Zofia Kobylińska, Marek Biesiadecki, Mateusz Mołoń, Sabina Galiniak

**Affiliations:** 1 Faculty of Medicine, University of Rzeszów, al. Tadeusza Rejtana 16C, 35-959 Rzeszów, Poland; pkielar@ur.edu.pl (P.K.); mbiesiadecki@ur.edu.pl (M.B.); 2 Faculty of Biology, Natural Protection, and Sustainable Development, University of Rzeszow, al. Tadeusza Rejtana 16C, 35-959 Rzeszów, Poland; zosiakob@icloud.com

**Keywords:** antioxidants, *Cistus*, disease prevention, extract, oxidative stress

## Abstract

For centuries, traditional medical systems have utilized *Cistus* leaf infusions, extracts, and essential oils in the treatment of inflammatory conditions, respiratory infections, febrile illnesses, and gastrointestinal disorders. Contemporary research has increasingly focused on the identification and characterization of biologically active constituents—particularly polyphenols and other antioxidants—that may modulate key physiological and cellular processes in the human body. These include mechanisms related to oxidative stress, inflammation, aging, and carcinogenesis. The therapeutic relevance of *Cistus*-derived compounds is further supported by their generally favorable safety profile and high tolerability, which distinguishes them from many synthetic pharmaceuticals. Moreover, the accessibility of *Cistus* preparations as dietary supplements or herbal infusions allows for their regular consumption without the need for complex therapeutic regimens. This positions *Cistus* as a promising candidate for integrative health strategies aimed at disease prevention and health maintenance. This review provides a comprehensive overview of the pharmacological potential and therapeutic applications of *Cistus* extracts, with particular emphasis on their antioxidant and bioactive properties.

## 1. Introduction

The *Cistaceae* family is a medium-sized group of plants within the order Malvales, comprising eight genera: *Cistus*, *Crocanthemum*, *Fumana*, *Halimium*, *Helianthemum*, *Hudsonia*, *Lechea*, and *Tuberaria*. In total, the family includes approximately 180 species of shrubs and herbaceous plants [1]. Among them, the genus *Cistus* (commonly known as rock rose) represents the largest and most widespread group, encompassing between 20 and 25 species. These plants are perennial flowering shrubs predominantly distributed throughout the Mediterranean region [2]. The taxonomy of the genus *Cistus* has undergone numerous revisions over time. Historical classifications reported varying numbers of species, ranging from 16 species according to Grosse (1903) to 28 species according to Dunal (1824) [3,4]. The difficulty in establishing a definitive number of taxa is primarily due to the high degree of polymorphism and the intensive hybridization processes occurring within the genus [2,5]. As a result, numerous hybrids have been identified within *Cistus*, such as *Cistus incanus* (English: hoary rock rose), which is a natural hybrid of *C. albidus* and *C. crispus*. Additionally, several subspecies have been described, including *C. creticus* subsp. *creticus*, *C. creticus* subsp. *eriocephalus*, and *C. creticus* subsp. *corsicus* [1,5,6].

Traditionally, the taxonomic classification of the genus *Cistus* was based on morphological characteristics, including both vegetative and reproductive traits. Key features considered included leaf shape and pubescence, number and arrangement of veins, flower structure, presence of glandular trichomes, and fruit size [1,3,4,7]. However, due to the high variability of these traits, numerous and often inconsistent intraspecific classifications were proposed. It was not until the development of advanced research methods, particularly the application of molecular analyses, that more precise determination of phylogenetic relationships within the genus became possible. These advancements led to the recognition of 21 accepted *Cistus* species [2,8]. The application of comparative analysis of nuclear DNA and chloroplast DNA sequences has enabled the delineation of three distinct subgenera within the genus *Cistus*: *Cistus*, *Leucocistus*, and *Halimioides* [1,7]. This integrative molecular approach has significantly enhanced the precision of taxonomic classification and contributed to a more comprehensive understanding of phylogenetic relationships within the family. The diversity of climatic and edaphic conditions within this region results in a species distribution pattern that reflects environmental gradients. The subgenus *Halimioides* (comprising three species) is restricted to the western Mediterranean region, whereas the subgenera *Leucocistus* (eight species) and *Cistus* (nine species) are distributed throughout the Mediterranean Basin and the Canary Islands [3,7,9].

The adaptation of the genus *Cistus* to Mediterranean environmental conditions is reflected in a suite of ecological traits, including fire-stimulated seed germination, insect-mediated pollination, and a reproductive cycle synchronized with the spring season [10]. A key aspect of the biology of these plants is their capacity to produce a large number of seeds that exhibit high thermal resistance, enabling rapid germination in the subsequent growing season. Additionally, species within the genus *Cistus* synthesize essential oils and resins that are highly flammable, often contributing to the propagation of wildfires in Mediterranean ecosystems [2,3,6,7,11].

Species of the genus *Cistus* have a natural ability to form hybrids due to cross-fertility. Additionally, the subgenus *Cistus* L. can form rarer and sterile hybrids with the subgenus *Leucocistus* [12]. *C. incanus* L., which is commonly used in natural medicine as an infusion and dietary supplement, is a hybrid of two species, *C. albidus* L. and *C. crispus* L. [13].

The aim of this narrative review is to provide a comprehensive and up-to-date overview of the phytochemical composition and biological activities of *Cistus* species, with particular emphasis on their antioxidant, antimicrobial, anti-inflammatory, and cytoprotective properties. By summarizing both in vitro and in vivo findings, this review seeks to highlight the therapeutic potential of *Cistus*-derived extracts and compounds, identify gaps in current knowledge, and propose directions for future research and clinical application.

A structured literature search was performed using electronic databases including PubMed, Scopus, Web of Science, and Google Scholar. Articles published between 2000 and 2024 were considered, with a focus on studies reporting on the following: chemical composition (e.g., flavonoids, terpenes, tannins, essential oils), in vitro and in vivo antioxidant, anti-inflammatory, antimicrobial, and cytoprotective effects, traditional and ethnomedical uses of *Cistus* species, safety, toxicity, and clinical trials related to Cistus-based preparations.

The following search terms (used singly and in combination) were applied: Cistus, rock rose, Cistus incanus, Cistus ladanifer, polyphenols, antioxidant activity, ethnopharmacology, anti-inflammatory, essential oil, traditional medicine, medicinal plants. Studies were included if they involved the use of Cistus plant material or extracts (aqueous, alcoholic, essential oils, fractions), reported experimental data (chemical profiling or biological testing), and were written in English or Polish. Review articles, in silico analyses, and traditional medicine surveys were used to support context and provide comprehensive background but were not the primary source of data for biological activity.

Data were extracted and grouped into thematic sections covering phytochemistry, antioxidant potential, anti-inflammatory and antimicrobial effects, antiglycation activity, and traditional applications.

## 2. Biology of *Cistus* sp.

### 2.1. Ecology and Geographic Distribution

**Figure 1:**
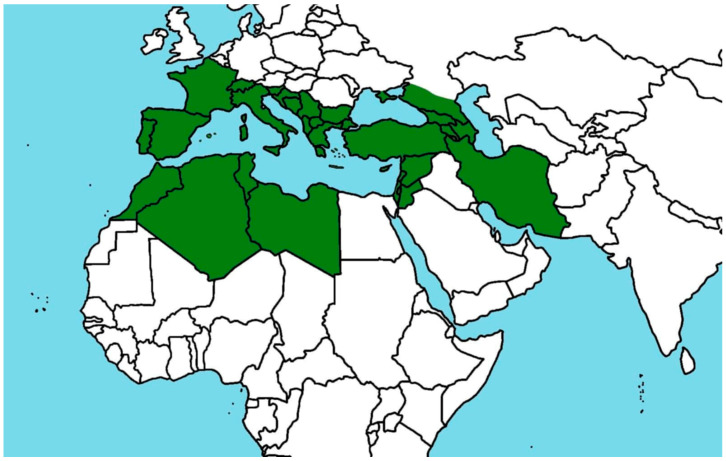
Occurrence of *Cistus* sp. The green color indicates the occurrence of *Cistus* sp.

Species belonging to the genus *Cistus* are primarily distributed throughout the Mediterranean Basin, although their range extends from the Canary Islands and Madeira, across the Mediterranean region, to the Caucasus, Israel, and northern Africa [1,11]. These plants are considered pioneer species, capable of colonizing early successional habitats, particularly in areas affected by soil degradation resulting from overgrazing, wildfires, or other environmental disturbances [2,3]. The distribution of selected species from the genus *Cistus* is presented in Table 1 and Figure 1.

*Cistus* plays a key ecological role in the formation of maquis plant communities, which are characteristic of Mediterranean ecosystems. These shrubs form dense thickets on dry, nutrient-poor, sun-exposed, and rocky soils, most commonly on calcareous substrates. They are also frequently found in mountainous regions, growing on both acidic and alkaline soils, particularly within oak and pine forests, at elevations reaching up to 1200 m above sea level [9,16]. Most species of this genus occur over quite large areas, but several of them are known as endemics [2].

### 2.2. Morphology

Plants of the genus *Cistus* are perennial, evergreen, woody shrubs that typically reach heights ranging from 50 to 150 cm. They are characterized by an erect stem with numerous, densely branched lateral shoots [1,7,11]. The lateral branches bear opposite or alternate, simple leaves up to 8 cm in length. These leaves may be flat or undulate, petiolate or sessile, and their surfaces are often covered with trichomes—an important diagnostic feature for species identification. These trichomes may be stellate or simple in form and play a crucial role in the production of essential oils and oleoresin, known as labdanum, which imparts a distinctive fragrance to the plants [1,11]. The flowers of *Cistus* species are bisexual, actinomorphic, and hypogynous, typically comprising three or five sepals, with the outer sepals usually smaller than the inner ones. The corolla consists of three to five petals, displaying a wide range of colors—from white to deep pink and purple—depending on the subgenus. Species within the subgenus *Cistus* bear purple-hued flowers, whereas members of the subgenera *Leucocistus* and *Halimioides* produce white flowers. In some species, distinctive dark red spots are present at the base of the petals [2,7,11]. The flowers are ephemeral, opening in response to morning light and remaining open for only a short duration [1,11]. Inflorescence structure within the genus *Cistus* is variable. Some species exhibit racemose or cymose inflorescences, forming racemes, umbels, or fan-shaped clusters, while in others, due to reduction, flowers may occur singly [11]. The androecium consists of numerous fertile stamens, and the single pistil is composed of an ovary formed by three to five carpels, although in *C. ladanifer*, the number may reach up to twelve [7,11]. These plants are predominantly self-incompatible, promoting cross-pollination both within and between species. Hybrid identification in natural habitats is relatively straightforward, as hybrids typically exhibit intermediate morphological traits between the parental species [3]. They produce a large number of polyhedral seeds with two cotyledons, which are highly resistant to elevated temperatures [11]. The roots of *Cistus* species form close symbiotic associations with various mycorrhizal fungi, primarily from the genus *Lactarius* [11].

## 3. Chemical Composition of *Cistus* sp.

Plants of the genus *Cistus* are renowned for their rich and complex chemical composition, encompassing a wide array of organic compounds with significant biological, ecological, and pharmacological relevance. The chemical profile of these plants exhibits considerable variability, influenced by multiple environmental factors such as temperature, solar radiation, humidity, and soil conditions [6]. Moreover, the chemical specificity of *Cistus* varies across species and geographic locations, which in turn affects its biological properties and potential applications [2].

These plants are particularly rich in secondary metabolites, including flavonoids, terpenes, phenolic acids, essential oils, and resins. Over 700 distinct chemical compounds have been identified in *Cistus*, among them were approximately 400 terpenes (including monoterpenes, sesquiterpenes, and diterpenes), around 150 polyphenols, various organic acids, and other bioactive constituents [11]. This diverse chemical composition underlies the broad spectrum of biological activities exhibited by *Cistus*, including antioxidant, antibacterial, and anti-inflammatory effects, making it highly valuable for pharmaceutical, cosmetic, and phytotherapeutic applications [1].

### 3.1. Phenolic Compounds

One of the key groups of chemical constituents in *Cistus* are phenolic compounds, which include flavonoids, tannins, and phenolic acids. Studies on the genus *Cistus* have identified as many as 72 different flavonoids, including 14 quercetin glycosides, 9 kaempferol glycosides, and 5 derivatives of myricetin. Among the tannins, 38 distinct hydrolyzable tannins have been characterized, known for their anti-inflammatory and antibacterial activities. Furthermore, 21 different phenolic acids have been detected, with caffeic, ellagic, and chlorogenic acids being the most prevalent—each recognized for their antioxidant and protective effects [11,18,19]. Flavonoids, which serve essential biological functions, include quercetin, kaempferol, and flavan-3-ols [6,20]. Additionally, catechins, gallocatechins, and proanthocyanidins are present, all of which exhibit potent antioxidant and protective properties [6]. From these catechins (-)-gallocatechin was the dominating compound in *C. incanus* samples in a study by Jeszka-Skorwon et al. [21]. Moreover, young leaves of *C. ladanifer* produce significantly higher levels of flavonoids and diterpenes than mature leaves or stems, showing clear seasonal variation. Plants younger than one year release notably fewer compounds. Notably, studies indicate that flavonoid concentrations in *Cistus* leaves peak during the summer months, underscoring their critical role in photoprotective mechanisms that enable the plant to withstand intense solar exposure [1,11]. Differences in secondary metabolite content related to plant part, age, and season likely influence the species’ interactions with both biotic and abiotic environmental factors [22]. Polyphenol metabolism plays a protective role against both biotic and abiotic stressors, such as by facilitating nitrogen retention in the soil, which supports plant growth in nutrient-poor environments [23,24]. The presence of these compounds is also associated with UV protection, enhancing the plant’s adaptation to harsh environmental conditions. Moreover, numerous phenolic compounds are involved in ecological interactions, such as attracting pollinators, thereby influencing the reproductive success and dispersal of the species [11,25].

### 3.2. Terpenes

Terpenes represent one of the key groups of secondary metabolites found in many plant species, including *Cistus*, playing essential biological roles such as protection against pathogens, participation in plant defense mechanisms, and influencing plant attractiveness to pollinators [26]. The terpene composition varies significantly depending on the species and subspecies. For instance, the essential oil of *C. ladanifer* is dominated by 1,8-cineole (19.27%) and viridiflorol (16.38%), which contribute to its intense, fresh aroma. In contrast, *C. monspeliensis* is characterized by a more complex, balsamic scent profile, with 1,8-cineole (9.14%), bornyl acetate (3.14%), and α-pinene (5.84%) as the major constituents [6]. The essential oil of *C. creticus* ssp. *corsicus* is rich in diterpenes, giving it a more resinous character, whereas *C. creticus* ssp. *eriocephalus* is dominated by sesquiterpenes, which impart a stronger, earthier aroma [5]. Variations in terpene profiles are likely the result of adaptations to differing environmental conditions, such as soil type, sunlight exposure, temperature, and water availability [2]. Gas chromatography–mass spectrometry (GC-MS) revealed variability in yield (0.19–0.42 mL/100 g) and composition, dominated by oxygenated sesquiterpenes and monoterpenes in *C. ladanifer* oils from 12 locations differing in soil type [27]. Notably, essential oil samples collected during the fruit ripening stage contained higher levels of monoterpenes and sesquiterpenes (including both hydrocarbon and oxygenated forms) compared to those from the flowering stage, which were richer in diterpenes and compounds categorized as ‘other’ in a recent study by Pérez-Izquierdo et al. [28]. Studies have shown that terpenes present in *Cistus* exhibit antibacterial, antifungal, and antiviral properties, which may explain their potential in pharmaceutical and cosmetic applications [20].

### 3.3. Essential Oils

All *Cistus* species accumulate essential oils, which constitute a significant component of their chemical profile. The content of these oils can vary depending on genotype, plant age, environmental conditions, and the method of raw material storage [1]. More than 120 different volatile compounds have been identified in *Cistus* essential oils, including numerous monoterpenes and sesquiterpenes. In *C. creticus* subsp. *corsicus*, the predominant volatile constituents are 13-epi-manoyl oxide (18.5%), manool (7.2%), and labda-7,14-dien-13-ol (3.8%) [5]. In *C. ladanifer*, viridiflorol (16.38%) and 1,8-cineole (19.27%) are the major components, while in *C. monspeliensis*, significant amounts of α-pinene (5.84%) and bornyl acetate (3.14%) have been detected [11]. In a separate study, El Hachlafi et al. used GC-MS analysis to reveal that the essential oil of *C. ladanifer* is primarily composed of linderol (17.76%), γ-terpinene (17.55%), and borneol (13.78%) as its major bioactive constituents [29]. GC-MS analysis conducted by El Karkouri et al. identified viridiflorol (17.64%), pinocarveol (11.02%), bornyl acetate (9.38%), and ledol (8.85%) as the principal constituents of *C. ladanifer* essential oil [30]. Chemical analysis of *C. albidus* L. essential oil showed camphene as the dominant compound (39.21%), with notable amounts of α-pinene (19.01%), bornyl acetate (18.32%), tricyclene (6.86%), and melonal (5.44%) also present in the composition [31]. Due to their high content of aromatic compounds, *Cistus* essential oils are highly valued in the perfume industry and are also used in aromatherapy and pharmaceutical formulations [1].

### 3.4. Oleoresin—Labdanum

Labdanum is a natural oleoresin (~70% of the resin) obtained from plants of the genus *Cistus*, particularly from species such as *C. ladanifer*. This substance serves as an important secretory product, fulfilling protective and adaptive functions for the plant, while also being a valuable raw material in the perfume industry due to its intense, resinous–balsamic fragrance and fixative properties [32]. Labdanum is a complex mixture of chemical compounds, primarily terpenoid and phenolic in nature. The main classes of compounds present in this resin include labdane-type diterpenes, methylated flavonoids, and phenylpropanoids [33]. GC-MS analyses showed that the diterpenoid fraction constitutes about 75% of the absolute extract and the flavonoid fraction about 15% [33]. The composition of *Cistus* oleoresin is influenced by species differences, environmental factors, and the extraction technique used, all of which can significantly affect its chemical profile and aromatic characteristics [34]. Chemical analyses conducted to date have confirmed the presence of numerous compounds already known from the literature, as well as novel, previously undescribed constituents. The complexity of labdanum’s chemical profile highlights its potential as a source of bioactive substances and as a valuable ingredient in the cosmetic and aromatherapeutic industries [32,33]. In cosmetics, labdanum extracts are used in skin care products for their anti-aging, antioxidant, and UV-protective effects, as it showed a spectrophotometric sun protection factor near 5, which is mainly due to flavonoids [33]. Moreover, labdanum resin derived from *C. ladanifer* L. holds potential as a source of bioactive compounds with anti-diabetic, neuroprotective, and antiproliferative properties [32]. However, the accumulation of essential oils and resins increases the flammability of these plants, which, in combination with high temperatures and intense solar radiation, often contributes to the occurrence of wildfires [6].

## 4. Biological Effects of *Cistus* Species: Targeting Oxidative Stress, Glycation, Inflammation, and Cancer

### 4.1. Traditional and Ethnopharmacological Applications of Cistus Species

It is currently estimated that approximately 25% of modern therapeutic drugs are derived from natural sources [30]. The presence of such compounds in contemporary pharmacology is rooted in their long-standing use in traditional medicine across various cultures. Among the plants of particular significance in this context are species of the genus *Cistus*, which have played an important role for centuries in traditional medicine of the Middle East and the Mediterranean Basin [35]. Plant materials—primarily aerial parts such as leaves, stems, and herbaceous shoots—have been valued for their rich chemical composition, which underlies their broad therapeutic applications [1,6,11].

According to ethnopharmacological data, various *Cistus* species, including *C. ladanifer* and *C. albidus*, have been used in the treatment of numerous ailments. In the Middle East, particularly in traditional Turkish medicine, *Cistus*-based preparations were commonly employed to treat inflammatory skin conditions, rheumatic diseases, and nephritis. The plant was also appreciated for its anti-ulcer, antidiarrheal, and wound-healing properties [1,6,36]. In Mediterranean countries such as Greece, Italy, and Spain, *Cistus* was traditionally used to treat gastrointestinal disorders, including diarrhea and ulcers, and serves as an antispasmodic agent [37]. Moreover, in folk medicine, cistus infusions were used to treat not only diarrhea, but also colds, including fever, and skin diseases [38]. Ethnobotanical observations from these regions indicate a wide range of uses, including treatment of bacterial and fungal infections, heart diseases, spleen disorders, osteoarthritis, neuralgia, and general fatigue [1,11]. Furthermore, *Cistus*-based preparations have been utilized in the treatment of liver and colon dysfunctions, respiratory conditions—such as upper respiratory tract infections and pertussis—as well as in the management of neurological and psychological disorders, including anxiety, insomnia, neuralgia, and muscle spasms [11,32,38]. These traditional uses provide a valuable foundation for modern pharmacological investigations into the therapeutic potential of *Cistus* species.

### 4.2. Oxidative Stress and the Therapeutic Potential of Cistus Species

Oxidative stress arises when the generation of reactive oxygen species (ROS) exceeds the capacity of antioxidant defense mechanisms, resulting in cellular damage and playing a key role in the development of various chronic diseases [39,40]. It is recognized as a major contributor to the onset and progression of metabolic disorders, cardiovascular diseases, cancer, and neurodegenerative conditions, including Alzheimer’s and Parkinson’s disease [41,42,43]. In this context, there is growing interest in the use of natural antioxidants as supportive agents in preventing or alleviating oxidative stress-related damage.

Plants from the genus *Cistus* (rock rose), particularly *C. incanus* and *Cistus creticus*, are rich in polyphenolic compounds with high antioxidant potential. These include flavonoids, phenolic acids, and ellagitannins, which have been extensively studied for their ability to modulate oxidative stress-related pathways [44,45].

The antioxidant activity (AA) of *Cistus* herb extracts is influenced by several factors, including the extraction method, the analytical assay used, and the geographic origin of the plant material [46,47]. For example, Bernacka et al. [18] found that water infusions of *C. x incanus* from Turkey exhibited higher AA than those from Greece and Albania, a variation attributed to genetic and environmental factors [48]. In another study, *C. salviifolius* showed the highest total polyphenol content (TPC) and AA among several *Cistus* species [49]. Similarly, *C. ladanifer* (121 mg caffeic acid equivalents/g dry weight) and *C. salviifolius* (105 mg caffeic acid equivalents/g dry weight) demonstrated particularly high TPC values [47]. Methanolic extracts of *C x incanus* leaves also exhibited high total antioxidant capacity (TAC), indicating their potential for use in dietary supplementation [50]. Furthermore, *C. ladanifer* and *C. salviifolius* demonstrated the highest AA in another study, with median values of 301 mg and 261 mg Trolox equivalents per gram dry weight, respectively [47]. In DPPH assays, *C. monspeliensis* showed greater radical scavenging efficiency than *C. incanus* [51]. Ethanol extracts of *C. creticus* exhibited strong AA, with IC_50_ values ranging from 7.85 ± 0.94 µg/mL [52] to 165.10 µg/mL [53], reflecting differences in extract composition. Similarly, ethanolic extracts of *C. ladanifer* showed significant antioxidant potential (IC_50_ = 266.6 ± 0.828 µg/mL), along with high reducing power in FRAP tests [54]. Notably, bioavailable samples of *Cistus* extracts displayed weaker antioxidant and metal-reducing activities than their non-digested or post-gastric counterparts [55].

Oxidative stress can lead to DNA damage, including strand breaks and base modifications, which are implicated in aging, mutations, and cellular dysfunction. Extracts from *C. laurifolius* (root, branch, and leaf) protected DNA from hydroxyl radical-induced damage [56]. Additionally, aqueous extracts of *C. incanus* and *C. monspeliensis* prevented the formation of linear DNA under UV irradiation in the presence of H_2_O_2_ [51]_._ Furthermore, ethanol extracts of *C. creticus* showed protective effects against oxidative DNA damage in vitro by reducing H_2_O_2_-induced plasmid DNA strand breaks, likely due to polyphenolic radical-scavenging activity [53].

Lipid peroxidation is a hallmark of oxidative stress, often assessed by measuring malondialdehyde (MDA) levels. *Cistus* extracts have been shown to significantly reduce MDA concentrations in various models. For instance, *C. incanus* and *C. monspeliensis* reduced lipid peroxidation in rat liver microsomes, with *C. monspeliensis* demonstrating greater efficacy at low concentrations [51]. A clinical study reported a 16% reduction in MDA (from 20 ± 5.5 to 15 ± 4.9 µmol/L; *p* < 0.01) after six weeks of *C. incanus* supplementation, with no further changes after another six weeks [57]. Additionally, essential oils derived from *Cistus* species have demonstrated the ability to suppress lipid peroxidation, as evidenced in the β-carotene bleaching assay [58]. Extracts of *C. creticus* also reduced lipid oxidation and extended the shelf life of meat products, supporting their use as natural antioxidants in food preservation [52]. The findings of a study by Jerónimo et al. [59] indicate that dietary inclusion of *C. ladanifer* enhances the α-tocopherol content in lamb muscle tissue, which in turn improves the meat’s oxidative stability by reducing lipid peroxidation. This suggests that *C. ladanifer* can serve as a natural antioxidant source in animal feed, potentially improving meat quality and shelf life. The results of a recent study by Tsolakou et al. [60] suggest that supplementation with a standardized formulation including *C. aurantium* and *C. creticus* led to a significant 26.72% decrease in triglyceride levels after 12 weeks, with non-significant improvements in high-density lipoprotein, total cholesterol, and low-density lipoprotein. These findings suggest a beneficial effect of *Cistus* on lipid metabolism.

Oxidative stress also impairs proteins by oxidizing amino acid residues, altering tertiary structures, and inhibiting enzyme activity, thereby disrupting cellular homeostasis. Protein oxidation, often measured by the accumulation of carbonyl groups, is a recognized marker of oxidative stress. Polyphenols from *Cistus* extracts inhibit ROS-induced protein oxidation. A study reported an 18% reduction in advanced oxidation protein products (from 66 ± 18 to 53 ± 17 µmol/L; *p* < 0.001) after six weeks of *C. incanus* supplementation, with no further change thereafter [57]. However, no significant effect on paraoxonase-1 activity was observed during the study period [57]. Interestingly, *C. monspeliensis* root extract enhanced mitochondrial function by increasing ATP production and catalase activity, highlighting its protective role against oxidative mitochondrial damage [61]. In addition, *C. monspeliensis* demonstrated a remarkable capacity to accumulate heavy metals without visible toxicity symptoms, likely due to efficient detoxification and enhanced antioxidant defenses, including elevated superoxide dismutase and peroxidase activities [62]. Studies on *Cistus albidus* under prolonged drought stress revealed the activation of enzymatic antioxidant defenses, including increased levels of peroxidases and superoxide dismutase. Proteomic analyses further showed redox modifications of proteins, suggesting that reversible oxidative changes regulate metabolic functions during both stress and recovery phases [63].

Glycation is a non-enzymatic process in which reducing sugars react with proteins, resulting in the formation of advanced glycation end products (AGEs), which are closely linked to oxidative stress and the development of diabetic complications [64]. *Cistus* extracts have shown notable antiglycation properties. For instance, *C. ladanifer* essential oil caused significant inhibition of hemoglobin glycation, with maximum activity at a concentration of 0.5 mg/mL [29]. Moreover, *C. salviifolius* and *C. monspeliensis* demonstrated strong AA, ferric-reducing power, and inhibitory effects against α-glucosidase and α-amylase, suggesting potential as therapeutic agents in managing hyperglycemia [65]. All aqueous extracts of *Cistus* species showed concentration-dependent enzyme inhibitory activity, with *C. salviifolius* extracts outperforming the reference compound quercetin in α-glucosidase inhibition [55]. Moreover, bioavailable aqueous extracts showed lower antiglycation activity than non-digested samples, though *C. salviifolius* retained the highest inhibitory activity among tested samples [55]. Furthermore, infusions of *C. incanus* and its components have demonstrated the ability to trap methylglyoxal, a precursor of AGEs [18].

**Figure 2:**
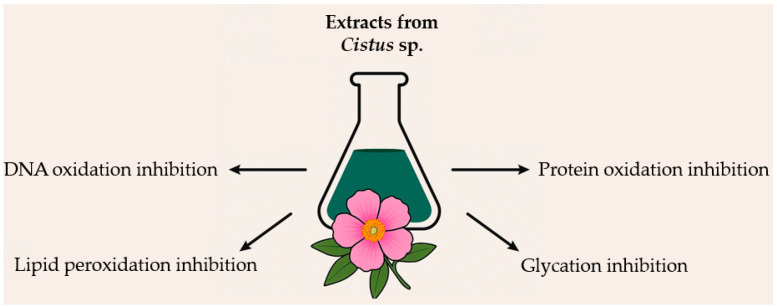
Summary of the effects of *Cistus* extract on biomolecular damage caused by oxidative stress.

The antioxidant and antiglycation effects of *Cistus* sp. underscore their potential as natural agents in managing oxidative stress and mitigating molecular damage. These plants effectively reduce lipid, protein, and DNA oxidation, enhance antioxidant capacity, and inhibit glycation processes (Figure 2). Although much of the current evidence stems from in vitro and animal studies, emerging data suggest promising applications of *Cistus*-based preparations in oxidative stress-related health interventions. However, further research is needed to confirm these effects in well-designed clinical trials. Additionally, the standardization of *Cistus* extracts and assessment of their bioavailability remain key challenges that should be addressed to ensure the consistent efficacy and safety of *Cistus*-based preparations.

### 4.3. Cellular and Molecular Insights into the Antioxidant and Anti-Inflammatory Effects of Cistus Extracts

The strong antioxidant properties of *Cistus* sp. are thought to be mediated by several biochemical mechanisms. These include, for example, direct scavenging of ROS and chelation of transition metal ions that catalyze oxidative reactions. Moreover, molecular docking analysis revealed that phenolic compounds from the *C. laurifolius* extract exhibit high binding affinity to both pro- and anti-apoptotic proteins (Bax, Bcl-2, Bcl-xl, Bad, and caspase-9), supporting their potential impact on oxidative stress and cell death [66]. Furthermore, an ethyl acetate fraction of *C. x incanus* leaves, enriched in myricetin and quercetin derivatives, demonstrated significant anti-inflammatory effects in lipopolysaccharide-stimulated RAW 264.7 macrophages. The extract reduced NO and PGE2 production, suppressed the expression of pro-inflammatory mediators such as interleukin (IL)-6 and cyclooxygenase-2, and enhanced IL-10 expression. Importantly, ethyl acetate fraction restored the nuclear translocation of nuclear factor erythroid 2-related factor 2 and upregulated its downstream target heme oxygenase-1, suggesting a protective mechanism involving redox-sensitive pathways. These effects are believed to be mediated by synergistic interactions among polyphenolic constituents, particularly myricitrin and rutin [67]. In terms of anti-inflammatory activity, essential oil extracted from the aerial components of *C. albidus* displayed a substantial lipoxygenase inhibition at 0.5 mg/mL [31]. Hydroalcoholic extracts from *C. laurifolius*, *C. salviifolius*, and *C. creticus* also exhibited notable anti-inflammatory activity. In particular, *C. laurifolius*, which contained the highest amount of quercetin among the tested species, showed the strongest effect in reducing nitrite levels (a marker of nitric oxide production) and significantly decreased PGE_2_ levels, indicating effective inhibition of pro-inflammatory pathways. Additionally, all three species reduced IL-6 levels in a concentration-dependent manner, further supporting their anti-inflammatory potential [49].

### 4.4. Anticancer Properties

The anticancer mechanism of *Cistus* extracts is primarily based on several complementary biological activities, including antioxidant, pro-apoptotic, antiproliferative, and anti-inflammatory effects (Table 2). Due to the presence of numerous bioactive compounds, such as polyphenols and flavonoids, *Cistus* extracts can reduce oxidative stress, induce programmed cell death in cancer cells, inhibit their proliferation, and mitigate inflammation associated with the carcinogenic process. In vitro studies showed that *Cistus* extract significantly reduced intracellular ROS levels and inhibited the growth of human breast (MCF-7) and colon cancer (LOVO) cell lines, particularly in drug-sensitive sublines by 15–28%, while sparing normal fibroblasts—highlighting its selective cytotoxicity and potential role as an adjuvant in cancer therapy [68]. Nevertheless, extracts from aerial parts and roots of *C. monspeliensis* also exhibited antioxidant potential, with root extracts showing stronger effects, evidenced by the increased viability of SH-SY5Y neuroblastoma cells exposed to H_2_O_2_-induced oxidative stress [61]. *C. monspeliensis* extract also exhibited concentration-dependent cytotoxicity in Chinese hamster ovarian K1 (CHO-K1) cells, with no significant effects observed below 75 µg/mL and an IC_50_ value of 228 µg/mL, indicating a threshold for safe biological application. The *C. monspeliensis* extract appears to act through antioxidant and antigenotoxic pathways, likely involving free radical scavenging, membrane stabilization, and interference with DNA damage signaling, particularly at non-cytotoxic doses [69]. Moreover, hexane extract of *C. monspeliensis* demonstrated notable antiproliferative activity against human melanoma A-375 cells, with an IC_50_ of 52.44 ± 3.69 µg/mL at 72 h, surpassing the efficacy of the reference drug 6-mercaptopurine. This effect is likely linked to the extract’s high content of polyunsaturated fatty acids, flavonoids, and vitamin E, which contribute to its antioxidant and cytotoxic properties [70]. Furthermore, leaf extract of *C. laurifolius* significantly increased TAC in colorectal (Caco-2) and breast (MCF-7) cancer cell lines in a dose- and time-dependent manner, confirming its potent antioxidant properties. Additionally, significant reductions in total oxidant capacity and oxidative stress index were observed, particularly after 24 h of exposure, indicating effective neutralization of ROS under oxidative stress conditions [66]. Furthermore, *C. laurifolius* has demonstrated cytotoxic and pro-apoptotic effects on MCF-7 breast cancer cells in vitro, with high-dose, prolonged exposure reducing cell viability, inhibiting DNA synthesis, and inducing apoptosis [71]. Based on the study by Gaweł-Bęben et al., *C. incanus* and *C. ladanifer* extracts exhibit promising anticancer activity, particularly against human melanoma cells (A375). The extracts, especially those of *C. incanus*, demonstrated cytotoxic effects with IC_50_ values as low as 57.8 µg/mL, while maintaining lower toxicity toward noncancerous keratinocytes. The anticancer potential is likely due to the high content of polyphenolic compounds such as epicatechin, epigallocatechin gallate, and myricitrin, which may act synergistically to induce cytotoxicity in melanoma cells [72]. Leaf extracts of *C. ladanifer* exhibit significant antiproliferative activity against human liver (HepG2), prostate (22Rv1), and breast (MDA-MB-231) cancer cell lines in a dose-dependent manner. The highest activity was observed with the hexanic extract on prostate cancer cells (IC_50_ = 11.32 µg/mL), suggesting that flavonoid-rich fractions may be particularly effective in targeting hormone-sensitive cancers [54]. A recent study by Guzelmeric et al. demonstrated that hydroalcoholic extracts of *C. laurifolius*, *C. salviifolius*, and *C. creticus* had potent anticancer effects against human pancreatic cancer cells (MIA PaCa-2) in both 2D and 3D models. Notably, *C. laurifolius* extract at 1 mg/mL led to a 98% reduction in spheroid size, indicating high selective cytotoxicity toward cancer cells with minimal effect on healthy dermal fibroblasts [49].

The increasing focus on phytochemicals with therapeutic potential has positioned polyphenols and flavonoids as promising candidates in oncological research. In response to this trend, recent studies have evaluated the influence of sterilization and encapsulation procedures on the chemical stability and bioactive retention of *Cistus* extract. These technological interventions aim to enhance the efficacy and reproducibility of formulation strategies. Experimental findings indicate that the preserved compounds exhibit potent cytotoxic effects against glioblastoma cell lines, while maintaining a favorable safety profile in non-tumorigenic cells, underscoring their potential as selective anticancer agents [73].

### 4.5. Antimicrobial Properties

**Figure 3:**
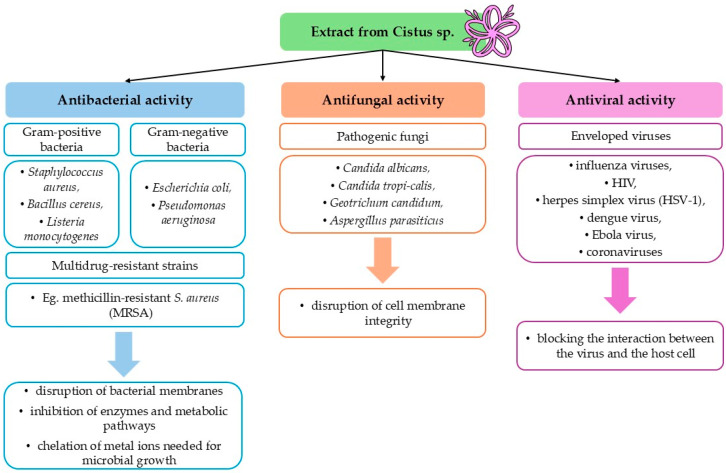
Summary of the antimicrobial properties of *Cistus* extract.

Plants of the genus *Cistus* exhibit a broad spectrum of biological activities, with particularly notable antimicrobial effects. This activity is attributed to the presence of numerous secondary metabolites, whose concentrations are key determinants of the plant’s therapeutic potential [11]. Their activity against drug-resistant strains and multiple pathogen types supports further investigation into their use as natural therapeutics or adjuncts in antimicrobial therapy (Figure 3).

#### 4.5.1. Antibacterial Activity

Numerous in vitro studies have confirmed the high efficacy of extracts from species such as *C. incanus*, *C. creticus*, *C. ladanifer*, and *C. monspeliensis* against a wide range of pathogenic bacterial strains. This activity encompasses both Gram-positive bacteria (e.g., *Staphylococcus aureus*, *Bacillus cereus*, *Listeria monocytogenes*) and Gram-negative bacteria (e.g., *Escherichia coli*, *Pseudomonas aeruginosa*) [71,74,75,76,77]. The ethanolic extract of *C. ladaniferus* exhibited antibacterial activity against both Gram-positive and Gram-negative bacteria, notably due to its high content of gallic acid and rutin, with inhibition zones up to 17 mm against *Bacillus subtilis* [78].

Of particular interest is the activity of *Cistus* extracts against multidrug-resistant strains, including the methicillin-resistant *S. aureus* (MRSA), for which conventional antibiotic therapies are increasingly ineffective [79,80]. The methanolic extract of *C. salviifolius* exhibited significant antistaphylococcal activity against MRSA strains, with inhibition zones reaching 13 mm and minimum inhibitory concentrations as low as 4 mg/mL [79].

Both aqueous infusions and hydroalcoholic extracts show activity, but ethanol-based extracts tend to be more potent due to the higher solubility of phenolics [78]. Minimum inhibitory concentrations reported for ethanolic or aqueous extracts often range from 50 to 200 μg/mL, depending on extract type and bacterial strain [75,81]. Aqueous and hydroalcoholic extracts of *C. salviifolius* exhibited the strongest bacteriostatic activity against *S. aureus* among four tested species, with minimum inhibitory concentrations values as low as 45–52 µg/mL, independent of total phenolic content, and are robust across drying methods [82]. Synergistic effects have been reported when *Cistus* extracts are combined with conventional antibiotics [83,84]. The antibacterial activity is believed to result from multiple mechanisms: disruption of bacterial membranes by flavonoids and terpenoids, inhibition of bacterial enzymes and metabolic pathways, and chelation of metal ions needed for microbial growth [85,86].

#### 4.5.2. Antifungal Activity

Current data indicate the effectiveness of *Cistus* extracts—particularly ethanolic and chloroformic extracts—against pathogenic fungi such as *Candida albicans*, *Candida tropicalis*, *Geotrichum candidum*, and *Aspergillus parasiticus* [29,87,88,89]. A study by Karim et al. [87] showed that aqueous extracts of multiple *Cistus* species, particularly *C. creticus, C. populifolius*, and *C. ladanifer*, achieved over 99% inhibition of *Geotrichum citri-aurantii* spore germination and reduced the incidence of citrus sour rot to as low as 8.33% in artificially inoculated mandarin fruit, indicating strong potential as natural postharvest fungicides.

The active compounds present in *Cistus* extracts, especially gallic acid, are believed to disrupt fungal cell membrane integrity, leading to increased permeability and loss of cellular homeostasis [17,19].

#### 4.5.3. Antiviral Activity

In addition to antibacterial effects, *Cistus* species also demonstrate significant antiviral potential, particularly against enveloped viruses. Rather than acting through conventional mechanisms of replication inhibition, *Cistus* extracts primarily function by blocking the interaction between the virus and the host cell. This mode of action has been observed in the context of influenza viruses (H7N7, H1N1, and H5N1), HIV, herpes simplex virus (HSV-1), dengue virus, Ebola virus, and certain coronaviruses [75,90,91]. The water–alcoholic extract of *C. incanus* completely inhibited both the extracellular virions and intracellular replication of herpes simplex virus SvHA 1 and SvHA 2 (including acyclovir-resistant strains) as well as human coronavirus HCoV-229E, with a selectivity index above 10 and up to 4-log reductions in viral titers, indicating strong potential as a broad-spectrum antiviral agent [92]. Furthermore, the aqueous extract of *C. incanus* inhibited a wide spectrum of HIV-1 and HIV-2 clinical isolates—including drug-resistant strains—by targeting viral envelope glycoproteins, preventing viral attachment and fusion, and also blocked entry of pseudotyped Ebola and Marburg viruses [90].

Unlike many antiviral drugs that target specific stages of the viral replication cycle, polyphenolic complexes in *Cistus* extracts can act directly on the viral envelope, leading to its destabilization and preventing infection of host cells [92]. This non-specific antiviral mechanism is particularly valuable in the context of RNA virus variability and their rapid development of resistance.

### 4.6. From Infusions to Pharmaceuticals: Multifunctional Uses of Cistus Extracts

The species most commonly used in the food and pharmaceutical industries for the production of herbal infusions and dietary supplements is *C. incanus* L. As a raw material, both plant materials (mainly leaves) and extracts are used [21,93,94,95]. Aqueous extracts of *C. ladaniferus* leaves and stems exhibit dose-dependent antispasmodic effects on isolated rabbit and rat jejunum, likely via calcium channel blockade. The activity was not altered by adrenergic receptor antagonists, supporting a non-adrenergic mechanism. These findings validate the plant’s traditional use for intestinal discomfort [96]. Furthermore, *C. salviifolius* L. and *C. monopoieses* L. aqueous extracts also possess anti-inflammatory properties as well as central and peripheral analgesic effects [97]. Similarly, recent in vitro studies have demonstrated that *C. laurifolius* extract may exert neuroprotective effects in a H_2_O_2_-induced neurodegeneration model using differentiated SH-SY5Y cells. Pre-treatment with the extract improved cell viability and significantly upregulated the neuronal marker MAP2, suggesting a protective effect against oxidative, stress-induced neuronal damage [98]. Extracts from the aerial parts of the plant can also be used during the occurrence of psychological stress by blocking the CRH-R1 receptor, which activates pathways transmitting signals of the body’s response to stress. Additionally, due to their anti-inflammatory and anti-aging properties, they can alleviate the effects of neurogenic stress, appearing, among others, on the skin, which may be important for people suffering from chronic stress [99]. Furthermore, *C. ladanifer* exhibits notable anti-inflammatory and wound-healing properties, primarily linked to its flavonoid and tannin content. Aqueous extracts from the aerial parts have shown significant activity in vivo, with both oral and topical applications reducing inflammation and promoting wound contraction in animal models. These findings support its therapeutic potential in skin and inflammatory disorders [99]. Extracts from *C. incanus* and C. *ladanifer*, particularly those rich in polyphenols such as myricitrin and epigallocatechin gallate, exhibit strong antioxidant, anti-tyrosinase, and photoprotective properties. These findings highlight their potential as multifunctional cosmetic ingredients for skin protection against photooxidative stress, hyperpigmentation, and UV-related damage [72]. Aqueous extracts of *C. incanus* have demonstrated anti-ulcer activity in rat models, showing dose-dependent protection against lesions induced by ethanol, hydrochloric acid, indomethacin, serotonin, and reserpine. The extract, rich in bioflavonoids, was particularly effective in reducing mucosal damage from serotonin and reserpine, likely by preserving gastric microvascular integrity [100]. *C. ladaniferus* possesses antihypertensive properties, which are mainly due to an endothelium-dependent vasodilatory action [101]. Some studies on *C. incanus* L extracts, both in mice and humans, show anti-influenza properties and effectiveness in treating symptoms of upper respiratory tract infections [102]. *C. monspeliasis* and *C. parviflorus* showed notable enzyme inhibition, particularly *C. monspeliensis* roots against acetylcholinesterase and butyrylcholinesterase, suggesting their utility as enzyme inhibitors in pharmaceutical and nutraceutical development [19].

## 5. Research Limitations and Future Perspectives

Despite the growing body of literature on the phytochemical richness and bioactivity of *Cistus* species, several limitations should be acknowledged. Firstly, the composition and efficacy of *Cistus* preparations vary significantly depending on species, plant part, harvest season, extraction method, and solvent used. This heterogeneity limits the comparability and reproducibility of results across studies. Secondly, although many biological activities have been demonstrated in vitro and in animal models, there is a notable scarcity of well-designed human clinical trials to validate safety, efficacy, and optimal dosing. Moreover, the molecular mechanisms underlying the observed effects—particularly antimicrobial, neuroprotective, and anti-inflammatory actions—remain incompletely characterized. Finally, chronic toxicity, potential herb–drug interactions, and the metabolic fate of major constituents (e.g., ellagitannins and diterpenes) are rarely investigated.

Future studies should focus on the development of standardized *Cistus* extracts with defined phytochemical profiles to ensure reproducibility and facilitate clinical translation. Establishing chemical fingerprints and validated markers (e.g., ellagitannins or diterpenes) are essential for therapeutic applications. Well-designed human clinical trials are needed to evaluate efficacy, safety, and bioavailability, particularly in the context of respiratory infections, metabolic disorders, and inflammatory conditions.

## 6. Conclusions

The genus *Cistus*, widely distributed across the Mediterranean Basin, offers a rich source of bioactive compounds with significant antioxidant, antimicrobial, anti-inflammatory, and cytoprotective potential. This review highlights the diverse phytochemical composition of *Cistus* species, particularly flavonoids, tannins, terpenes, and essential oils, and summarizes their biological activities across various models. Notably, *Cistus* extracts have demonstrated efficacy in modulating oxidative stress, inhibiting microbial growth, and supporting metabolic and neurological health. While in vitro and in vivo data are promising, further standardized clinical trials are needed to validate therapeutic claims. The growing interest in *Cistus* as a natural agent supports its continued investigation for pharmaceutical, cosmetic, and functional food applications.

## Figures and Tables

**Table 1:** List of species of the genus *Cistus* and their natural occurrence.

Species	Natural Occurrence	References
*C. albanicus* Heywood	Albania, Greece	[14]
*C. albidus* L.	Mediterranean region (Canarian), Iberia, France, Italy, North Africa, Corsica, Sardinia	[4]
*C. asper* Demoly & R. Mesa	Canarian region	[15]
*C. atlanticus* (Humbert & Maire) Demoly	Morocco	[15]
*C. atriplicifolius* Lam.	Morocco, Spain	[15]
*C. calycinus* L.	Morocco, Portugal, Spain	[15]
*C. chinamadensis* Bañares & P. Romero	Canarian region	[15]
*C. clusii* Dunal	Algeria, Baleares, Italy, Morocco, Portugal, Sicilia, Spain, Tunisia	[15]
*C. creticus* L.	central-eastern Mediterranean (Corsica, Sardinia), Morocco	[15]
*C. crispus* L.	Endemic in France, Spain, the Iberian and Apennine Peninsulas, and northwest Africa	[16]
*C. grancanariae* Marrero Rodr. & al.	Canarian region	[15]
*C. halimifolius* L.	Canarian region	[15]
*C. heterophyllus* Desf.	Spain, North Africa	[15]
*C. horrens* Demoly	Canarian region	
*C. inflatus* Demoly	France, Portugal, Spain	[15]
*C. ladanifer* L.	western Mediterranean region	
*C. lasianthus* Lam.	France, Morocco, Portugal, Spain	[15]
*C. laurifolius* L.	Mediterranean mountains	[17]
*C. libanotis* L.	Portugal, Spain, Argelia	[15]
*C. monspeliensis* L.	western Mediterranean to the Canary Islands and Madeira	[4,11]
*C. munbyi* Pomel	Algeria, Morocco	[15]
*C. ocymoides* Lam.	Morocco, Portugal, Spain	[15]
*C. osbeckiifolius* Webb	Canarian region	[15]
*C. palmensis* Bañares & Demoly	Canarian region	[15]
*C. parviflorus* Lam.	eastern Mediterranean, Greece, Turkey, Italy, Cyprus, Libya	[15]
*C. populifolius* L.	France, Morocco, Portugal, Spain	[16]
*C. pouzolzii* Delile	Algeria, Morocco, France	[15]
*C. psilosepalus* Sweet	Iberia, France	[15]
*C. salviifolius* L.	Mediterranean Basin	[4,11,17]
*C. symphytifolius* Lam.	Canarian region	[15]
*C. umbellatus* L.	Algeria, France, Greece, Lebanon–Syria, Morocco, Portugal, Spain	[15]

**Table 2:** Anticancer activity of *Cistus* sp.

Study Type	Species of *Cistus*	Model	Key Findings	References
in vitro	*C. laurifolius*	human pancreatic cancer cells (MIA PaCa-2)	anticancer effects, indicating high cytotoxicity selectively toward cancer cells with minimal effect on healthy dermal fibroblasts	[49]
in vitro	*C. salviifolius*	human pancreatic cancer cells (MIA PaCa-2)	anticancer effects	[49]
in vitro	*C. creticus*	human pancreatic cancer cells (MIA PaCa-2)	anticancer effects	[49]
in vitro	*C. ladanifer*	human liver (HepG2) cancer cell lines, human prostate (22Rv1) cancer cell lines, breast (MDA-MB-231) cancer cell lines	antiproliferative activity	[54]
in vitro	*C. monspeliensis*	neuroblastoma cells (SH-SY5Y)	increased viability of cells exposed to H_2_O_2_	[61]
in vitro	*C. laurifolius*	colorectal (Caco-2) cell lines, breast (MCF-7) cancer cell lines	increased TAC, reduction in total oxidant capacity and oxidative stress index	[66]
in vitro	*C. incanus*	human breast (MCF-7) cell lines, colon cancer (LOVO) cell lines	reduced intracellular ROS levels and inhibited the growth	[68]
in vitro	*C. monspeliensis*	Chinese hamster ovarian K1 (CHO-K1) cell	cytotoxicity	[69]
in vitro	*C. monspeliensis*	human melanoma A-375 cells	antiproliferative activity	[70]
in vitro	*C. laurifolius*	breast (MCF-7) cancer cell lines	cytotoxic and pro-apoptotic effects	[71]
in vitro	*C. incanus*, *C. ladanifer*	human melanoma cells (A375)	anticancer activity, cytotoxic effects	[72]

## Data Availability

No new data were created or analyzed in this study.
